# The Freiburg framework for multimodal *ex situ* assessment of neural plasticity in human cortical tissue

**DOI:** 10.3389/fnsyn.2026.1771781

**Published:** 2026-03-16

**Authors:** Jakob Straehle, Christos Galanis, Lukas Grünewald, Elli-Anna Balta, Tobias D. Deller, Ute Häussler, Boris Mizaikoff, Jürgen Beck, Andreas Vlachos

**Affiliations:** 1 Department of Neurosurgery, Medical Center, Faculty of Medicine, University of Freiburg, Freiburg, Germany; 2 Center for Advanced Surgical Tissue Analysis (CAST), Faculty of Medicine, University of Freiburg, Freiburg, Germany; 3 Department of Neuroanatomy, Institute of Anatomy and Cell Biology, Faculty of Medicine, University of Freiburg, Freiburg, Germany; 4 Center BrainLinks-BrainTools, University of Freiburg, Freiburg, Germany; 5 Translational Epilepsy Research, Department of Neurosurgery, Medical Center, University of Freiburg, Freiburg, Germany; 6 Institute of Analytical and Bioanalytical Chemistry, Ulm University, Ulm, Germany; 7 Hahn-Schickard Institute for Microanalysis Systems, Ulm, Germany; 8 Center for Basics in Neuromodulation (NeuroModulBasics), Faculty of Medicine, University of Freiburg, Freiburg, Germany

**Keywords:** electrophysiology, human cortical tissue, microscopy, multimodal tissue analysis, optimized tissue handling, stimulated Raman histology, transcranial magnetic stimulation, translational neuroscience

## Abstract

Studying human cortical physiology requires access to viable brain tissue, yet species-specific differences limit the translational value of animal models. To address this, multiple laboratories have developed *ex situ* approaches for investigating neurosurgical access tissue using electrophysiological, molecular, and imaging techniques. Here, we introduce the Freiburg framework—a structured, multimodal approach that integrates high-resolution electrophysiology, advanced imaging, molecular analyses, and Raman microscopy to assess neuronal and glial function under controlled, near-native conditions. Clinical metadata, including preoperative MRI, together with in-patient controls is systematically incorporated to account for biological variability and to enable human-to-human translational (H2H) comparisons. The framework further enables controlled neuromodulatory and pharmacological interventions, including *ex situ* repetitive transcranial magnetic stimulation (rTMS). By formalizing an end-to-end experimental pipeline, the Freiburg framework supports systematic investigation of human-specific neurophysiological mechanisms and provides a robust foundation for translational human neuroscience.

## 1 Introduction

Human brain research remains a central challenge in neuroscience. Much of our knowledge of neural function, plasticity, and disease mechanisms derives from studies in rodents and non-human primates (Capitanio and Emborg, 2008; Verdier et al., 2015; Azkona and Sanchez-Pernaute, 2022). While these models have been indispensable for elucidating fundamental principles, their translational value is inherently constrained by interspecies differences in cytoarchitecture, molecular signaling, and circuit organization (Oberheim et al., 2009; Molnár et al., 2016; Beaulieu-Laroche et al., 2018; Boldog et al., 2018; Hodge et al., 2019; Campagnola et al., 2022; Loomba et al., 2022). Moreover, many neuropsychiatric and neurodegenerative disorders display human-specific pathophysiological features, limiting translation from preclinical findings (Granzotto et al., 2024; Hermann et al., 2025). Addressing these limitations requires direct investigation of human neural tissue under well-defined experimental conditions, an approach historically constrained by limited access to viable human brain samples for research.

An increasingly important strategy to overcome these constraints is the use of neurosurgical access material, which provides a unique opportunity to study living human brain tissue *ex situ*. During neurosurgical procedures, cortical or subcortical tissue may be removed to facilitate surgical access or optimize resection. When not required for diagnosis, this tissue is typically discarded in standard clinical practice. Repurposing neurosurgical access material enables direct assessment of human neuronal and glial function, synaptic physiology, and disease-associated cellular and molecular interactions. Unlike conventional animal-to-human translation, this approach supports human-to-human (H2H) *horizontal translation,* in which controlled comparisons are performed across individuals, brain regions, and experimental conditions within the human cortex. While this strategy offers a more direct route to understanding human-specific plasticity mechanisms, it also necessitates rigorous strategies to account for substantial inter- and intraindividual variability.

**Figure 1:**
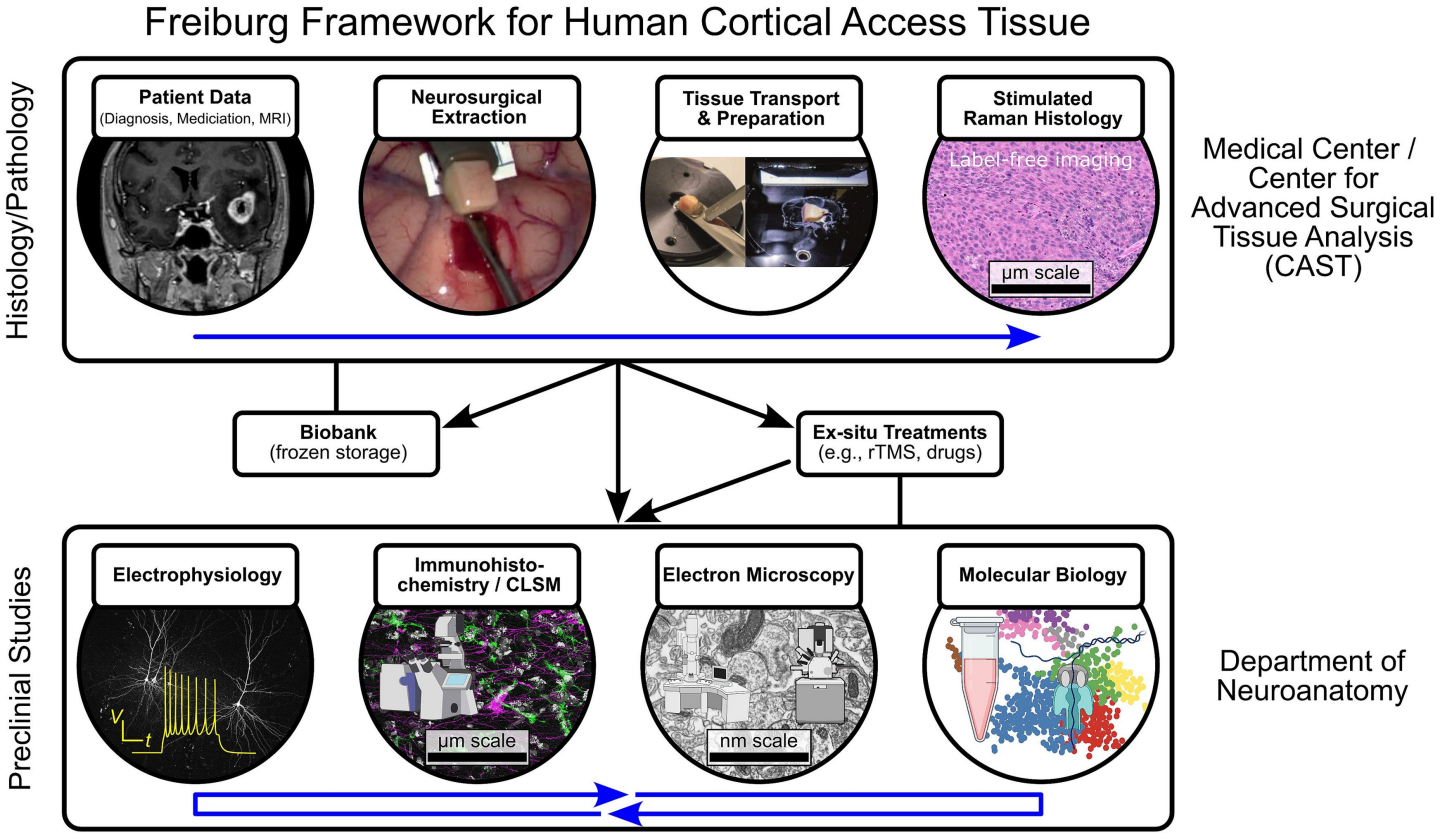
Schematic overview of the Freiburg framework for multimodal analysis of human cortical access tissue. The workflow integrates preoperative imaging, standardized tissue handling, and multimodal quality control to ensure the viability and interpretability of neurosurgical access material for preclinical research. Top row: Following MRI-guided neurosurgical resection, cortical tissue is immediately immersed in artificial cerebrospinal fluid (aCSF) and rapidly transported to the laboratory. After vibratome sectioning, a central quality-control step is performed using stimulated Raman histology (SRH). SRH enables rapid, label-free assessment of tissue integrity and tumor infiltration at the microscopic level, complementing preoperative MRI. Based on this multimodal evaluation, intact tissue is selected for biobanking, direct experimental analysis, or additional *ex situ* interventions (e.g., repetitive transcranial magnetic stimulation, rTMS). Samples are subsequently allocated to downstream applications, including electrophysiology, structural analyses (immunohistochemistry/confocal laser scanning microscopy and electron microscopy), molecular profiling (transcriptome/proteome), and organotypic slice culture. Patient-specific clinical metadata are retained throughout the workflow to enable correlation of experimental findings with clinical parameters. Elements of the schematic were adapted from (Straehle et al., 2023) (published under a Creative Commons Attribution 4.0 International License) and generated using Biorender, BioIcons, and NIAID NIH BioArt.

At the University of Freiburg, we have established a structured framework to harness neurosurgical access material for basic neuroscience research within the Center for Advanced Surgical Tissue Analysis (CAST) (Figure 1). This framework emphasizes rapid and standardized tissue processing, enabling transcriptomics (Ravi et al., 2022; Zhang et al., 2023), high-resolution electrophysiological recordings, and imaging techniques (Lenz et al., 2021, 2024; Rosado et al., 2022). As a specific example, this framework is used to address questions such as the structural correlates of rTMS-induced plasticity (detailed in Section 7). A distinctive feature of our approach is the integration of label-free stimulated Raman scattering (SRS) microscopy (Freudiger et al., 2008; Neidert et al., 2022; Straehle et al., 2022), which allows non-destructive, molecular-level assessment of tissue composition and tumor infiltration while preserving tissue integrity for downstream functional and ultrastructural investigations. The augmentation of SRS via correlated quantum cascade laser based mid-infrared imaging microscopy providing complementary molecular information is currently in progress. This multimodal approach bridges experimental and clinical neuroscience and establishes a foundation for systematic H2H translational studies of human cortical plasticity.

## 2 Ethical considerations and patient consent

The use of human neurosurgical access material for basic and translational research requires a well-defined ethical framework that balances scientific opportunity with patient autonomy and safety. At the University of Freiburg, tissue collection is conducted under protocols approved by the local ethics committee. All patients receive comprehensive information about potential research use and provide preoperative written informed consent. This process ensures transparency regarding data protection, sample anonymization, and future applications.

A core ethical principle of the Freiburg framework is that all tissue samples originate exclusively from resections performed for clinical indications, with no modification of surgical strategy for research purposes. Ethical procedures are aligned with international best-practice standards to promote responsible use of human tissue and to facilitate standardized, reproducible H2H translational research across institutions.

## 3 Surgical planning, MRI-guided tissue selection, and tissue handling

**Figure 2:**
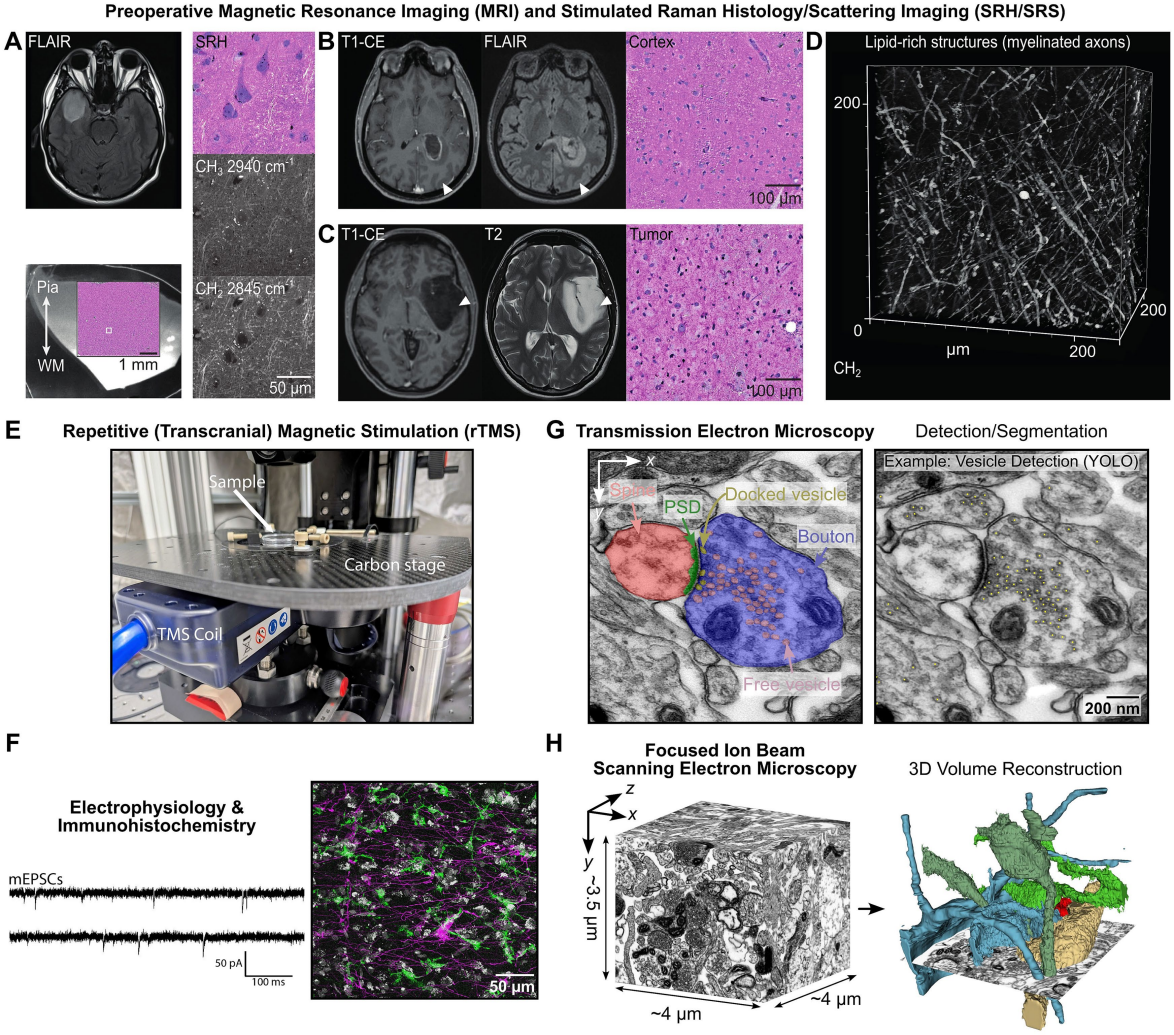
Multimodal characterization of human cortical access tissue from preoperative MRI and Raman-based imaging to functional and ultrastructural analyses. **(A)** Cortical access tissue from resection of a right temporal IDH-mutant astrocytoma (WHO grade 2). Tumor marked by high intensity in the FLAIR signal. Lower left panel shows a 250 μm tissue slice undergoing stimulated Raman histology (inset). Right panel: pseudo H&E-like stimulated Raman histology (SRH). Imaging was performed using NIO microscope (Invenio Imaging, Santa Clara, USA). Visualization of nuclei, myelinated axons, and intracellular lipid-rich organelles. **(B)** Tumor-free neocortical access tissue obtained during surgical resection of an intraventricular glioblastoma (WHO grade 4). Arrowheads represent the location of cortical access tissue marked by neuronavigation (Stryker, Kalamazoo, USA). **(C)** Tumor-infiltrated access tissue derived from a non-contrast enhancing IDH-mutant astrocytoma (WHO grade 2). **(D)** Volumetric SRS imaging acquired in the CH_2_ stretching band, predominantly representing lipid-rich structures (myelinated axons). Cortical tissue originated from the parieto-occipital lobe and was resected during surgical treatment of pharmacoresistant epilepsy (focal cortical dysplasia type IIA). Imaging was performed using a Leica Stellaris CRS system (Leica, Wetzlar, Germany). **(E)** Experimental setup for repetitive (transcranial) magnetic stimulation and electrophysiology. **(F)** Exemplary miniature excitatory postsynaptic current (mEPSC) whole-cell patch clamp recording from which event frequencies and amplitudes are extracted. Representative confocal laser scanning microscopy image showing interaction of microglia (aIba1-green) and astrocytes (aGFAP-magenta) identified by immunohistochemical labeling. **(G)** TEM bright-field imaging provides high-resolution 2D ultrastructural information but is limited in axial (*z*) resolution due to section thickness (~60 nm, ZEISS LEO 906E). Synaptic features, including presynaptic boutons, free and docked synaptic vesicles, postsynaptic densities (PSD), and postsynaptic spines, can be reliably identified and quantified. Expert-labeled datasets derived from TEM and FIB-SEM images are used to train and refine automated segmentation pipelines based on deep learning approaches, for example, synaptic vesicle detection using YOLO-based object detection (Diwan et al., 2023). **(H)** FIB-SEM tomography provides (near-)isotropic voxel resolution with superior *z*-resolution compared to TEM and is used to generate accurate 3D reconstructions of synaptic and subcellular structures (Thermo Scientific Helios 5 CX). This complements TEM by enabling targeted volumetric imaging of selected regions within multimodal experimental workflows. A small volume of human cortical tissue and a few segmented neurons are shown (Fedorov et al., 2012).

The integration of neuroimaging is a central component of preoperative planning and intraoperative identification of neurosurgical access material (Figures 2A–C). Preoperative magnetic resonance imaging (MRI), including T2/FLAIR- (fluid attenuated inversion recovery) and T1-weighted contrast-enhanced (T1-CE) sequences facilitate the identification of non-eloquent cortical regions and enables assessment of the spatial relationship between access tissue and pathological structures like tumor infiltration zones and peritumoral edema (Würtemberger et al., 2022) (Figures 2B,C). Intraoperative MRI-based neuronavigation systems are used to document the location of the resected access tissue. While MRI-based measurements are readily integrated into the routine workflow and provide an essential macroscopic estimate of tumor proximity they are limited in detecting microscopic infiltration.

To address this limitation, we incorporate label-free stimulated Raman histology (Orringer et al., 2017) as a complementary, slice-level quality control step to assess tumor infiltration directly in the cortical access tissue (Figures 2A–C). In the near future, these measurements will be augmented by complementary and likewise label-free mid-infrared imaging analysis further expanding the molecular data space. This multimodal assessment enables classification of samples based on both radiographic context and molecular tissue composition, thereby supporting physiological investigations under near-native conditions.

Intraoperatively, cortical access tissue is excised following established microdissection principles to preserve sample integrity (Straehle et al., 2023). This minimizes mechanical stress, maintains cortical orientation, and avoids cauterization or suction. Immediately after resection, tissue is immersed in carbogenated, cold artificial cerebrospinal fluid (aCSF) to preserve electrophysiological and molecular properties for downstream analyses (Ting et al., 2018b; Lenz et al., 2021). Transfer time from operating room to the laboratory is minimized (≤15 min) to preserve tissue viability. Upon arrival, the tissue undergoes standardized vibratome sectioning. Subsequently, the tissue slices are allocated for various downstream applications, including acute electrophysiology, molecular profiling, ultrastructural analysis, and organotypic culture (Figure 1). Detailed protocols can be found in references (Ting et al., 2018b; Straehle et al., 2023; Zhang et al., 2023).

## 4 Stimulated Raman scattering microscopy for label-free tissue characterization

Stimulated Raman scattering (SRS) microscopy enables label-free molecular characterization of unfixed tissue by detecting vibrational signatures from inelastic light-matter interactions (Freudiger et al., 2008). Stimulated Raman histology (SRH) represents an imaging mode of SRS microscopy optimized for histopathological assessment by targeting specific molecular vibrations relevant to tissue architectures, such as CH_2_ or CH_3_ stretch modes (Orringer et al., 2017; Hollon et al., 2020). Processing of SRS signals generates histology-like images that closely resemble conventional hematoxylin–eosin (H&E) staining, enabling assessment of cytoarchitecture in unfixed tissue (Figure 2A) (Invenio Imaging Inc., Orringer et al., 2017). SRH has demonstrated clinical utility for intraoperative neuropathological diagnosis and the assessment of tumor margins, particularly in glioma surgery (Straehle et al., 2022; Hollon et al., 2023; Kondepudi et al., 2025).

Within the framework, SRH serves as a non-destructive quality control that complements preoperative MRI and informs downstream experimental analyses (Figures 2A–C). SRH enables μm-scale characterization of freshly resected cortical tissue, allowing detection of subtle structural alterations and regional heterogeneity. In particular, SRH can identify regions of interest for subsequent (ultra-)structural analyses, for example by visualizing axonal myelination patterns (Wang et al., 2005; Bélanger et al., 2009) (Figure 2D).

In practice, one 250 μm thick vibratome slice from each cortical access specimen is imaged prior to experimental use (Figure 2A). By combining SRH with electrophysiology, molecular profiling, and (ultra-)structural analyses, our workflow enables a robust, multimodal investigation of human neuronal and glial function under near-native conditions.

## 5 Ultrastructural analysis of human cortical tissue

Electron microscopy (EM) of human cortical tissue provides ultrastructural insights into synaptic organization, glial interactions, subcellular architecture, and the extracellular matrix. Recent advances (for review, see Titze and Genoud, 2016; Kievits et al., 2022; Kubota et al., 2025) have enabled nanoscale investigation of human cortical circuits (Shapson-Coe et al., 2024), confirming unique synaptic features specific to human cortex (Loomba et al., 2022). Within the Freiburg framework, EM complements functional and molecular readouts, enabling direct structure–function correlations.

To address the limited availability of human samples, we adopt a pragmatic, multi-resolution strategy that prioritizes targeted, hypothesis-driven EM analyses over exhaustive large-volume reconstructions (Shapson-Coe et al., 2024; Wu et al., 2024). This approach facilitates systematic comparison of multiple samples across patients, disease states, and experimental conditions, which is particularly relevant for investigating synaptic plasticity and treatment-induced structural changes (Lenz et al., 2021).

The core EM workflow focuses on small- to intermediate-scale (few μm to tens of μm), high-resolution analyses optimized for comparative studies. Conventional two-dimensional transmission electron microscopy (TEM) and three-dimensional serial-section TEM (ssTEM) (Rosado et al., 2022) are employed for rapid, quantitative assessment of synaptic features, including presynaptic boutons, postsynaptic spines, synaptic vesicles, and postsynaptic densities (Figure 2G). Image analysis combines expert manual annotation with machine-learning-based approaches, including object detection [e.g., YOLO (Diwan et al., 2023), Figure 2G] and segmentation [e.g., U-Net (Ronneberger et al., 2015)]. To complement TEM-based analyses, focused ion beam scanning electron microscopy (FIB-SEM) tomography (Knott et al., 2008; Narayan and Subramaniam, 2015) is applied to selected samples where high-resolution three-dimensional reconstructions are required (Figure 2H). FIB-SEM tomography provides superior axial (*z*) resolution compared to ssTEM, which enables detailed visualization of sub-synaptic structures (e.g., synaptic vesicle pools).

Access to additional EM techniques, including array tomography (Micheva and Smith, 2007) and serial block-face scanning EM (Denk and Horstmann, 2004; Motta et al., 2019; Loomba et al., 2022), is provided through the EMcore facility, allowing flexible scaling of ultrastructural analyses. Larger-scale EM experiments are conducted in collaboration with specialized centers, with the Freiburg framework contributing optimized tissue preparation and access to human cortical material. Overall, these ultrastructural analyses within our multimodal workflow are essential for quantifying the synaptic and subcellular architecture underlying human cortical plasticity.

## 6 Inter-individual differences and in-patient controls

A central challenge in human neuroscience is the inter-individual variability inherent to the human brain, with each individual exhibiting distinct cytoarchitectural, molecular, and functional characteristics. Genetic background, age, disease history, medication, and environmental influences all contribute to variability in neuronal and glial function. While animal models offer tightly controlled experimental systems, the heterogeneity of human cortical tissue necessitates dedicated strategies to ensure interpretability and comparability of experimental findings.

Empirical evidence underscores the importance of accounting for such variability. For example, we have shown that age significantly influences the structure and function of superficial pyramidal neurons in the adult human neocortex, with older individuals exhibiting reduced dendritic spine density while largely preserving excitatory synaptic transmission (Lenz et al., 2024). Clinical parameters further modulate neuronal function, as demonstrated by differences observed between patients with epilepsy receiving antiepileptic medication and patients with brain tumors treated with corticosteroids (Lenz et al., 2024). Incorporating clinical metadata is therefore essential for interpreting results from human cortical slices. Within the framework, we record clinical metadata including demographics (age, sex), clinical history (diagnosis, medication, radiation therapy), preoperative MRI characteristics, and intraoperative parameters (time-to-lab, resection location) (Moore et al., 2011). These can later be correlated with experimental findings and stored, e.g., along with images with tools such as OMERO (and similar for other data modalities).

In addition to inter-individual variability, intra-individual differences across cortical regions pose a further challenge. Neuronal and glial properties may vary between cortical areas, limiting the generalizability of findings derived from a single resected sample. Because neurosurgical resections are necessarily guided by clinical considerations, direct comparisons across multiple cortical regions within the same individual are often not feasible. To complement access-tissue-based studies, the Institute of Anatomy and Cell Biology, in collaboration with the Institute of Forensic Medicine, is exploring the use of postmortem human brain material for (ultra-)structural and molecular analyses. While postmortem tissue is not suitable for functional experimentation, it provides valuable spatial context and enables regionally resolved investigations that inform and contextualize findings obtained from living tissue.

For functional and perturbational studies in viable human cortical tissue, we implement in-patient controls as a core experimental design principle for *ex situ* interventions. Different slices from the same resected tissue are assigned to experimental and control conditions, enabling assessment of treatment effects against an internal baseline. This strategy minimizes confounding effects arising from both inter-individual and inter-regional variability and enhances the interpretability of *ex situ* experiments. Together, the integration of postmortem reference data and in-patient-controlled access-tissue experiments strengthen the Freiburg framework as a platform for robust H2H translational neuroscience.

## 7 Neuromodulation with transcranial magnetic stimulation

A central application of the Freiburg framework is investigating human cortical plasticity using repetitive (transcranial) magnetic stimulation (rTMS). rTMS is an established neuromodulatory technique capable of inducing lasting changes in cortical excitability and synaptic function and is widely applied in the treatment of neuropsychiatric disorders (Hallett, 2007; Wagner et al., 2009; Jannati et al., 2023). Despite its clinical efficacy, the cellular and synaptic mechanisms underlying rTMS-induced plasticity remain incompletely understood, particularly in the human cortex.

To address this, r(T)MS is applied to freshly resected human cortical tissue, enabling controlled interrogation of stimulation-induced effects on neuronal and glial physiology (Figure 2E). In contrast to *in vivo* stimulation, where network-level dynamics and compensatory mechanisms complicate interpretation, *ex situ* preparation allows dissociation of primary cellular and synaptic effects from secondary circuit-level responses. This controlled setting enables causal analysis of r(T)MS-induced plasticity at the level of individual cells and synapses.

To ensure physiological relevance and reproducibility, electric fields induced by r(T)MS in the tissue are estimated using finite element modeling (FEM) (Turi et al., 2021a,b; Schultheiss et al., 2025). These simulations guide optimization of coil positioning and stimulation parameters, ensuring that field strengths approximate those achieved in clinical and experimental *in vivo* applications. This makes FEM an integral component of the workflow, enabling standardized stimulation conditions across experiments and samples.

In addition to *ex situ* stimulation, the framework supports presurgical rTMS applied *in vivo*, followed by *ex situ* analysis of the stimulated cortical target regions. This approach allows direct investigation of cellular, molecular, and ultrastructural correlates of clinically relevant stimulation protocols. However, presurgical stimulation inherently limits experimental control, as inter- and intra-individual variability cannot be addressed to the same extent and in-patient controls become challenging when interventions are applied prior to surgery. Presurgical rTMS is therefore considered a complementary translational mode within the framework, trading experimental control for increased clinical proximity.

By integrating electrophysiological whole-cell patch clamp recordings (Figures 2E,F), we investigate r(T)MS-induced plasticity across functional, molecular, and structural scales. This multimodal approach enables identification of cellular and synaptic signatures associated with stimulation-induced changes, including alterations in synaptic efficacy, dendritic remodeling, and glial responses. Together, these analyses provide mechanistic insights into human rTMS-induced plasticity and establish a H2H translational platform linking clinical neuromodulation to fundamental neurophysiology.

## 8 Extending the framework: investigating other interventions and long-term tissue maintenance

Beyond rTMS, the Freiburg framework can accommodate a broad spectrum of invasive and non-invasive neuromodulatory and pharmacological interventions. Numerous stimulation modalities (Polanía et al., 2018), including transcranial direct current stimulation (tDCS) (Brunoni et al., 2012), focused transcranial ultrasound stimulation (TUS) (Darmani et al., 2022), deep brain stimulation (DBS) (Lozano et al., 2019), and related approaches, are known to induce plasticity at the systems level. However, their cellular and synaptic mechanisms of action in the human brain remain incompletely understood. The Freiburg framework provides a controlled experimental environment for systematic analyses at cellular, molecular, and ultrastructural scales.

Pharmacological modulation represents a complementary approach to probing and modulating human cortical plasticity. Using neurosurgical access material, we have previously demonstrated that all-trans retinoic acid (atRA) induces both structural and functional synaptic plasticity in human cortical neurons, characterized by increased excitatory synaptic strength and dendritic spine remodeling (Lenz et al., 2021). Combined with electrophysiological recordings, molecular profiling, and imaging, our framework enables systematic analysis of drug-induced plasticity mechanisms in the human cortex.

A further extension of this framework involves the long-term maintenance of viable human cortical tissue. Established protocols for organotypic human cortical slice cultures have demonstrated preserved cellular viability and network activity over periods ranging from several days to weeks (Verwer et al., 2002; Eugène et al., 2014; Ravi et al., 2019; Schwarz et al., 2019). However, slicing and prolonged *in vitro* cultivation are inherently associated with structural remodeling, particularly affecting long-range axonal projections. Associative, commissural, and projection fibers are necessarily severed during surgical resection and vibratome slicing, leading to progressive reorganization of axonal architecture during culture. As a consequence, long-term preparations preferentially preserve local microcircuit properties, whereas conclusions regarding intact large-scale connectivity and long-range network integration must be interpreted carefully.

To extend experimental time scales while maintaining controlled conditions, the Freiburg framework incorporates emerging approaches for chronic tissue maintenance, including microfluidic culture platforms (Low et al., 2021; Ingber, 2022). Such systems enable precise control of perfusion, oxygenation, and metabolic conditions, and can be combined with microelectrode arrays (MEAs) for long-term, non-invasive electrophysiological monitoring (Obien et al., 2015; Cerina et al., 2023). Integration of these platforms extend experimental timelines while maintaining near-native physiological conditions.

Long-term tissue viability further enables the application of genetic tools established in animal models. Viral vector-based gene delivery allows expression of genetically encoded reporters and sensors, facilitating investigation of neuronal and glial activity (Andersson et al., 2016; Ting et al., 2018a). These approaches provide a functional bridge between human and animal studies and enable the use of optogenetic actuators, calcium indicators, and voltage-sensitive probes in human cortical circuits. In addition, precise genetic perturbations, including CRISPR-based gene editing and RNA interference, offer new opportunities to interrogate disease-relevant molecular pathways in patient-derived tissue.

Manipulation and monitoring of human cortical circuits over extended time scales within our multimodal framework would represent a substantive advance for both basic neuroscience and translational research, with implications for neuromodulation, pharmacotherapy, and regenerative strategies.

## 9 Discussion: toward a standardized and collaborative framework

The study of human cortical physiology *ex situ* has gained substantial momentum, with research groups worldwide establishing increasingly sophisticated approaches to investigate neuronal and glial function in neurosurgical access material. While these efforts have generated important insights, the field remains methodologically fragmented, with substantial heterogeneity in tissue handling, stimulation paradigms, and data acquisition strategies (Hermann et al., 2025). Standardization of experimental procedures and harmonization of data collection are essential for enhancing reproducibility and comparability across studies. Establishing shared best practices encompassing neurosurgical resection, tissue processing, viability assessment, electrophysiological recording conditions, (ultra-)structural analyses, and genetic manipulation will enable cross-laboratory comparisons and cumulative progress in human neuroscience.

Beyond methodological harmonization, coordinated multi-center initiatives will be required to fully exploit the potential of *ex situ* human tissue research. These efforts enable systematic investigation of clinical variables (disease state, medication, neuromodulation) on synaptic plasticity and cellular resilience. Integrating standardized experimental pipelines with detailed clinical metadata will be critical for advancing human-to-human translational approaches and for identifying principles of plasticity that are specific to the human brain.

By fostering collaboration across institutions, the neuroscience community can establish a unified, human-centered framework for studying cortical plasticity that bridges basic research and clinical application. The Freiburg framework provides a structured and integrative foundation for this effort; its broader impact will depend on continued methodological convergence, transparent data-sharing practices, and sustained international collaboration.

## Data Availability

The data analyzed in this study are subject to the following licenses/restrictions: Data available upon reasonable request, subject to ethical approval. Requests to access these datasets should be directed to andreas.vlachos@anat.uni-freiburg.de.
